# Investigation of the Performance and Fuel Oil Corrosion Resistance of Semi-Flexible Pavement with the Incorporation of Recycled Glass Waste

**DOI:** 10.3390/ma18153442

**Published:** 2025-07-22

**Authors:** Ayman Hassan AL-Qudah, Suhana Koting, Mohd Rasdan Ibrahim, Muna M. Alibrahim

**Affiliations:** 1 Center for Transportation Research, Department of Civil Engineering, Faculty of Engineering, Universiti Malaya, Kuala Lumpur 50603, Malaysia; ay.qudah@yahoo.com (A.H.A.-Q.);; 2 Department of Architectural Engineering, Hijjawi Faculty of Engineering Technology, Yarmouk University, Irbid 21163, Jordan; muna.alibrahim@yu.edu.jo

**Keywords:** open-graded glass asphalt mixture, glass semi-flexible pavement, glass waste recycling, waste management, fuel oil corrosion resistance, appearance characteristics

## Abstract

Semi-flexible pavement (SFP) is a durable and cost-effective alternative to conventional rigid and flexible pavement and is formed by permeating an open-graded asphalt (OGA) layer with high-fluidity cement grout. The degradation of SFP mattresses due to fuel oil spills can result in significant maintenance costs. Incorporating glass waste (GW) into the construction of SFPs offers an eco-friendly solution, helping to reduce repair costs and environmental impact by conserving natural resources and minimizing landfill waste. The main objective of this research is to investigate the mechanical performance and fuel oil resistance of SFP composites containing different levels of glass aggregate (GlaSFlex composites). Fine glass aggregate (FGA) was replaced with fine virgin aggregate at levels of 0%, 20%, 40%, 60%, 80%, and 100% by mass. The results indicated the feasibility of utilizing FGA as a total replacement (100%) for fine aggregate in the OGA structural layer of SFPs. At 100% FGA, the composite exhibited excellent mechanical performance and durability, including a compressive strength of 8.93 MPa, a Marshall stability exceeding 38 kN, and a stiffness modulus of 19,091 MPa. Furthermore, the composite demonstrated minimal permanent deformation (0.04 mm), a high residual stability of 94.7%, a residual compressive strength of 83.3%, and strong resistance to fuel spillage with a mass loss rate of less than 1%, indicating excellent durability.

## 1. Introduction

Approximately 18 million tons of GW are generated annually worldwide following its extensive use in the modern industry and life. Disposal of such huge amounts of glass waste is a matter of concern because it is non-biodegradable and causes environmental contamination. However, due to its favorable physical properties and chemical composition, FGA has been successfully applied in the construction sector, particularly in road construction. The use of FGA in asphalt mixture as an aggregate replacement has been a hot research topic in the past decade. The Marshall stability test [1,2], mastic draindown test [3], skid resistance test, resilient modulus test [4], air voids test [3], moisture susceptibility test [1,2,4], indirect tensile strength test [5,6], and fatigue resistance test [7,8] have been conducted by many researchers to evaluate the performance and engineering properties of glass–asphalt mixtures. The incorporation of FGA in road construction offers significant engineering, environmental, and economic advantages when compared to conventional asphalt mixtures, including enhanced performance, improved traffic visibility, reduced CO_2_ emissions, lower energy consumption, and reduced material and landfill tipping costs [1,2,7]. This, in turn, indicates that GW can contribute to longer-lasting pavements with lower maintenance needs, thereby lowering overall life-cycle costs.

Depending on the structure, road pavement can be categorized into two primary types: rigid (concrete) and flexible (asphalt) where each type has many advantages and disadvantages [9]. As a feasible alternative to these conventional pavements, the SFP surface course (mattress) has recently been introduced. The SFP mattress is also known as grouted macadam composite or semi-rigid pavement, among scholars, highway authorities, and agencies. Most importantly, the structure and construction technology of all mentioned pavements are identical, consisting of the OGA structural layer with air contents of 20–35% [10,11,12,13], typically with a thickness of up to 6 cm. This porous layer is subsequently filled with high-fluidity cement grout. As a result, the SFP mattress retains some of the flexibility of asphalt materials, while exhibiting high surface rigidity that can serve as an economical and practical alternative to rigid pavements. Due to its good properties, SFP has been suggested as a superior mattress for complex environments or unfavorable traffic areas where there is likely to be frequent spillage of aggressive materials (petrol products), and for the renovation of heavy traffic areas that are heavy duty, or low speed, such as industrial areas, bus and parking terminal floors, loading platforms, airport aprons, and similar applications.

Since the SFP mattress is originally composed of two complex mixtures, asphalt and cement, its quality depends on the compositional materials of the two mixtures. Accordingly, the success of the SFP mattress as a high-quality pavement under heavy wheel loads and extremely harsh environmental conditions is primarily influenced by several key factors, such as asphalt content and type, aggregate gradation and properties, and air content within the OGA structure. Gradation has an important role in determining all pavement properties, including stability, mastic draindown, air voids, strength properties, skid resistance, stiffness, permeability, and resistance to moisture damage [9,14,15,16,17,18]. For the SFP mattress, an OGA layer with voids below the minimum allowable range (20%) can cause a retardation in grout penetration due to the presence of isolated voids throughout the depth of the OGA structure. This results in insufficient grout impregnation, which can adversely affect the quality of the following SFP mattress in terms of abrasion and strength properties. Moreover, the excess asphalt content in the OGA mixture can form a thick coating around the aggregate, resulting in reduced interfacial adhesion properties and accelerated abrasion.

Similarly, cement grout has a profound effect on the performance of the SFP mattress as it is in direct contact with the OGA surface under axial loads. Therefore, the grout must provide adequate strength to prevent abrasion and cracking. The main requirements for grout are its ability to rapidly penetrate the OGA structural layer via gravitational force without vibrations and to subsequently develop high strength to support the overall performance of the SFP mattress. Many factors, such as the content and type of cement [10], water/cement ratio [19], dosage and type of superplasticizer [20], sand size and type [12], and other admixtures [21], can significantly affect the performance of cement grout. Nevertheless, the construction of the SFP mattress leads to the use of large quantities of ordinary Portland cement (OPC), which causes significant emissions of CO_2_ and other greenhouse gases. Therefore, this study involved the application of FGA sand as a substitute to granite sand in cement grout to reduce such gases.

Spills of solvent liquid (i.e., fuel oils) onto SFPs and flexible pavements accelerate the deterioration process, leading to premature failures, increased lifecycle costs, and serious environmental impacts and traffic safety risks. The management of such spills requires effective containment and cleanup strategies to mitigate the effect. The structural behavior of SFPs more closely resembles the behavior of flexible pavements than rigid pavements [22]. This indicates the susceptibility of SFP mattresses to fuel oil spillage. Fuel oil cavitation pavement damage is defined as pavement degradation resulting from the interaction between the asphalt and fuel oils. This phenomenon is particularly prevalent in areas prone to fuel spillage, such as gas stations, parking lots, and during vehicle breakdowns.

The main fuel oils used in vehicle engines are gasoline and diesel, especially for heavy-duty vehicles. Due to their chemical similarity to asphalt, these fuel oils can dissolve asphalt binders, compromising the structural integrity of the pavement [12]. With increasing traffic volumes, fuel oil spillage from vehicles is inevitably worsening on roadways, especially involving poorly maintained heavy vehicles. Spilled oil dissolves the asphalt and causes the pavement surface to soften, weaken, and segregate, particularly under the combined effects of high temperatures, rainfall, and traffic loading. This degradation ultimately leads to pits and a number of corrosion-induced damage states, such as stripping, potholes, and raveling. Consequently, pavement durability, driving comfort, and skid resistance are significantly impaired [23,24].

A number of studies have addressed the resistance of asphalt mixtures to fuel oil corrosion. Li and Li [25] studied the change in mass of asphalt mixtures before and after the immersion process in oil as a damage degree indicator and the mechanisms of fuel corrosion resistance. Giuliani and Merusi [26] evaluated the resistance of airfield pavement to fuel corrosion by measuring changes in the viscoelasticity properties of asphalt. Meng et al. [27] examined the long-term effects and mechanisms of fuel-induced corrosion on the adhesive properties of asphalt. The study showed that fuel corrosion significantly weakens adhesion, promotes erosion, and leads to the formation of cracks. Additionally, spilled oil causes the loss of large molecular components from the asphalt binder, further degrading its performance. In another study, the principle of “likes-dissolve each other” was used to describe the mechanism of oil–asphalt corrosion [28]. A study conducted by Chaturabong et al. [29] demonstrated that the surfaces of dense-graded asphalt mixtures treated with tack coat and chip seal exhibited adequate skid resistance and can remedy oil spillage. In addition, many aspects of the effect of fuel oil spillage on the performance properties of asphalt mixtures, such as surface appearance, Marshall stability, Cantabro abrasion, skid resistance, compressive strength, indirect strength, resistance to moisture-induced damage, and wheel tracking have been studied [24,30,31,32]. Furthermore, asphalt mixtures treated with tack coat and chip seal [29], silicone resin [32], and asphalt modified with fuel-resistant modifier [33] as well as PE and SBS [34] were investigated. The findings showed that treated mixtures and modified asphalts always have a better resistance to oil corrosion in comparison with conventional mixtures and pure asphalt. On the other hand, the resistance of SFP composites to fuel corrosion was evaluated with a very narrow scope, and the literature review is inadequate in relation to this research topic. For example, Guo and Hao [35] pointed out that an SFP mattress has excellent resistance against oil corrosion due to its high density. Khan et al. [36] investigated the fuel corrosion resistance of SFP composite prepared from waste PET cementitious grouts and concluded that the mass loss of SFP composites due to fuel oil corrosion was negligible.

A study showed that SFP composites can be applied in areas frequently exposed to fuel spillage, such as fuel stations, oil refineries, and industrial floors [11]. Bharath et al. [37] demonstrated that the durability and structural integrity of the SFP composite remained high even after exposure to fuel oil corrosion. The results revealed that the retained tensile strength of the SFP composite after 24 h of immersion in a diesel bath was 92%, in comparison with 64% for a dense-graded mixture. According to Hao et al. [38], the test results of residual Marshall stability (RMS) showed that the SFP composite soaked in gasoline for 24 h had a higher fuel resistance compared to dense-graded asphalt mixtures, retaining 89% of its stability. Hirato et al. [39] also indicated that compared to asphalt mixtures, the RMS of SFP composite after 48 h immersion in kerosene exceeded 80%. Despite ongoing research in the field, the fuel oil corrosion resistance and performance of SFP composites incorporating GW as a fine aggregate replacement have not yet been reported. Accordingly, the present research is both timely and necessary, as it provides novel insights into the fuel oil corrosion resistance of SFP incorporating FGA. This contribution not only enhances understanding in a relatively underexplored domain but also supports the advancement of more sustainable and resilient pavement technologies.

The objective of this research was to investigate the mechanical performance and fuel oil corrosion resistance of sustainable glass-semi-flexible pavement composites grouted with glass cement grout. To accomplish this objective, a comprehensive experimental program was conducted, including degree of saturation, Marshall stability, indirect tensile stiffness modulus (ITSM), compressive strength, dynamic creep, visual appearance assessment, mass loss, residual stability, and residual compressive strength. Additionally, field emission scanning electron microscopy (FESEM) was employed to examine the internal morphology and micromechanical characteristics of the GlaSFlex composites.

## 2. Materials and Methods

### 2.1. Materials

**Figure 1:**
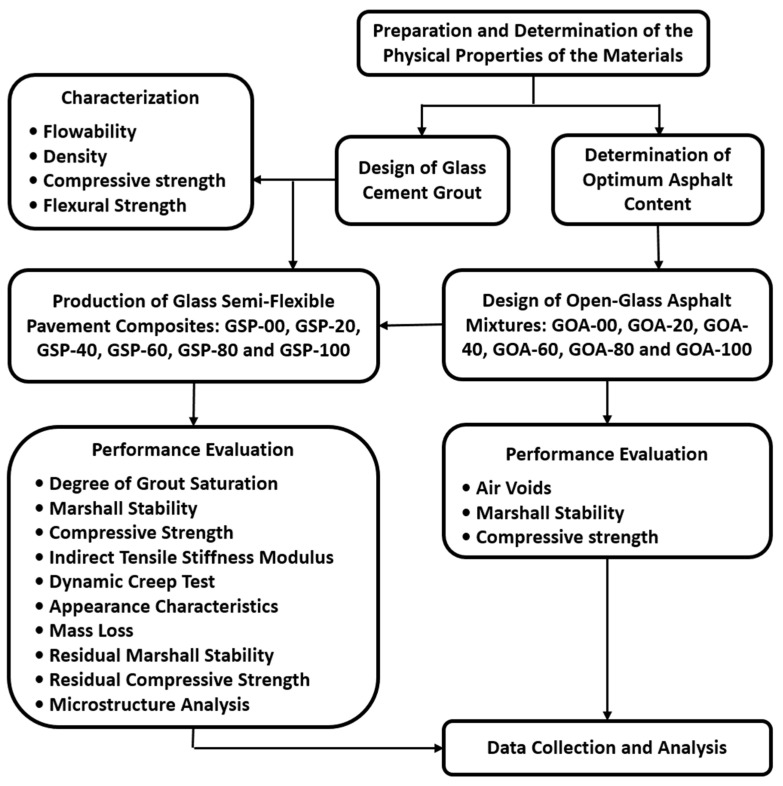
The experimental program of this research.

**Figure 2:**
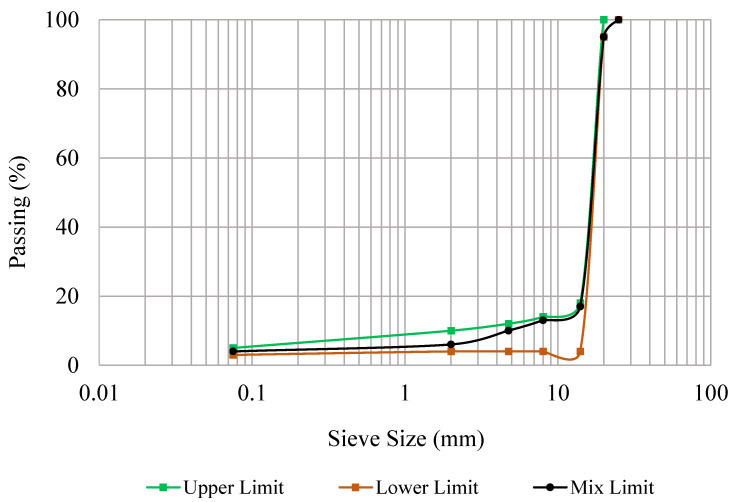
The grading curve of open-graded glass asphalt mixtures.

The experimental investigation scheme of this research is shown in Figure 1. All materials used in this research were sourced from local suppliers in Kuala Lumpur, Malaysia. The aggregates used in this study were granite aggregates obtained from the Quarry of Kajang. Table 1 shows the physical properties of aggregates. The aggregate gradation with a nominal maximum size of 25 mm was selected for the design of Glasphalt mixtures, which is adopted in Malaysia by the Road Engineering Association (REAM) [40]. The gradation is presented in Table 2 and plotted graphically in Figure 2. Asphalt of (60/70) grade used for the design of Glasphalt mixtures was obtained from Klang west refinery. The basic properties of asphalt are presented in Table 3.

FGA with a maximum fraction size of 2 mm was utilized as a fine aggregate replacement in the Glasphalt mixtures. It is important to note that FGA sand with a maximum size of 0.30 mm was introduced as part of the granite sand in the cement grout. The FGA material was produced only from GW bottles that were collected from a glass recycling facility in Petaling Jaya. Table 1 portrays the physical properties of FGA. OPC was also utilized in the Glasphalt mixtures, and the cement grout and its physical properties are presented in Table 4. Moreover, a superplasticizer with a density of 1.095 g/cm^3^ at 25 °C was introduced to reduce water content and promote hydration. All technical properties of the used materials satisfied the requirements of the REAM specifications [40].

In the fuel oil corrosion resistance tests, 95# gasoline, hereinafter referred to as RON95, was selected as the corrosion medium. This choice was based on the prevalence of traffic accidents involving small passenger cars, which often result in localized fuel spillage from damaged fuel systems. As RON95 is the most commonly used gasoline grade in such vehicles, its use provides a realistic simulation of fuel exposure conditions typically encountered on pavements in accident-prone or high-traffic areas.

### 2.2. Mixture Preparation Methods

#### 2.2.1. Open-Graded Glass Asphalt Mixtures

All asphalt mixtures were produced in accordance with the Marshall mix method [52]. This involved the production of compacted specimens in the form of a cylinder with a diameter of 101.5 ± 0.3 mm and a height of 69 ± 1. The asphalt content was 3.5% by mass of the total mix. To ensure that the mixture with 3.5% asphalt fulfilled the REAM requirements, three key parameters were evaluated: draindown, abrasion loss, and air void. According to the REAM standard [40], the acceptance criteria for OGA mixtures are an abrasion loss of less than 25%, a draindown of less than 0.30%, and an air content between 25% and 30%. At 3.5% asphalt, the mix met the required technical specifications, exhibiting an abrasion loss of 21%, an air void content of 26%, and a draindown rate of 0.12%.

In this study, six open-graded glass asphalt mixtures were prepared using different concentrations of glass as a replacement to fine aggregate (hereinafter referred to as Glasphalt mixtures). The FGA replacement levels were 0%, 20%, 40%, 60%, 80%, and 100% by mass. The mixture containing 0% FGA was used as the reference mixture. The mineral filler was replaced with OPC in order to reduce mastic draindown. All Glasphalt mixtures were produced by blending the asphalt binder with the aggregates. First, the combined aggregate fractions (FGA and granite) were heated at 160 °C for an hour. The asphalt was also heated for an hour at 150 °C and then added to the heated aggregates. The mixture was thoroughly blended at approximately 150 °C for 10 min to ensure a homogeneous mix. The mixture was transferred into a Marshall mold, and compacted using 50 blows/face at 145 °C. The compacted specimens were left in molds to cool in open air for 24 h. Table 5 summarizes the FGA concentrations in the asphalt mixtures, which are in consistent with the specifications defined by REAM [40].

#### 2.2.2. Cement Grout

**Figure 3:**
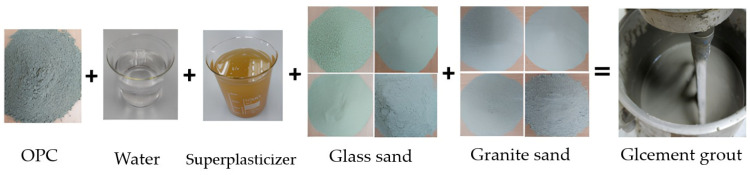
Materials used for the design of glcement grout.

The “glcement grout” in this study refers to the cement grout containing glass as a sand replacement. This grout was prepared using five materials, as presented in Figure 3. The composition of the glcement grout was determined through an experimental approach conducted in the laboratory. The sand to cement ratio was 30% (9% granite sand and 21% glass sand), which provides the best properties as reported by Saboo et al. [20]. The other material ratios were adopted due to the required liquidity to be achieved.

The formation of glcement grout was performed by mixing the dry materials (glass sand, granite sand, and OPC) for 3 min at a low speed. Next, 2/3 of the liquid materials (water and superplasticizer) were poured into the dry mix and then left to blend for 5 min at low speed. The remaining liquids were added, and the mixing process continued for approximately 7 min. This procedure enhances the workability of the grout. The mixing proportions and properties of the glcement grout are summarized in Table 6. All technical properties of the glcement grout met the requirements [40]. The results are agreement with Bayraktar’s [53] findings, which indicated that the addition of FGA and silica fume improves the strength of cement grout and can be used in buildings with high fire risk.

#### 2.2.3. Glass Semi-Flexible Pavement Composites

**Figure 4:**
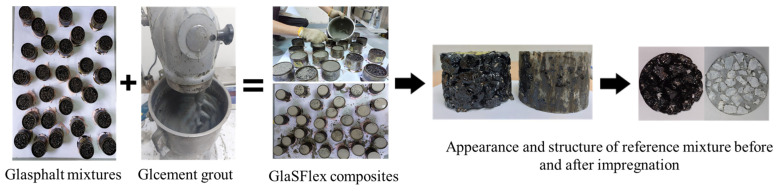
Photographic presentation of the production of GlaSFlex composites.

The “GlaSFlex composite” is defined as an open-graded glass asphalt mixture grouted with the glcement grout (see Figure 4). All Glasphalt specimens used in the fabrication of GlaSFlex composites were designed following the method described in Section 2.2.1. The designed specimens were left inside the molds; the undersides of the specimens were properly sealed to prevent the glcement leakage during impregnation. The specimens were first transferred to a flat surface, and the glcement grout was poured onto the top of the specimens and spread thoroughly with a rubber brush, as shown in Figure 4. No external vibrations were applied because the liquidity of the glcement grout was adequate. The GlaSFlex specimens were kept at ambient temperature and removed from the molds after 48 h of impregnation. The FGA concentrations in the SFP composites are listed in Table 5.

### 2.3. Performance Evaluation Methods

#### 2.3.1. Air Void Test

The air void content in the mixture is a very important parameter, as it allows the grout to penetrate the structure of the OGA mixture without the need for additional vibration. Furthermore, determining the air void content is essential for calculating the degree of grout saturation. Air voids were estimated in compliance with ASTM D3203 [54].

#### 2.3.2. Degree of Grout Saturation Test

A new concept adopted in SFP composites is the degree of grout saturation (D_st_), which serves as a key indicator of how well the mixture is grouted. Equation (1) is used to calculate the degree of grout saturation. The remaining air voids after impregnation should be in the range of 94–97% [55,56].

(1) $$D_{st}=100\%\times \left[\frac{\left(m_{1}-m_{2}\right)}{\left(V_{air}\times \rho \times V_{t}\right)}\right]$$

where D_st_ is the degree of grout saturation; m_1_ and m_2_ denote the Glasphalt mass before and after impregnation in g, respectively; V_air_ refers to the air content of Glasphalt mixture; ρ signifies the density of the glcement grout in g/cm^3^; and V_t_ represents the volume of compacted Glasphalt specimen in cm^3^.

#### 2.3.3. Marshall Stability Test

**Figure 5:**
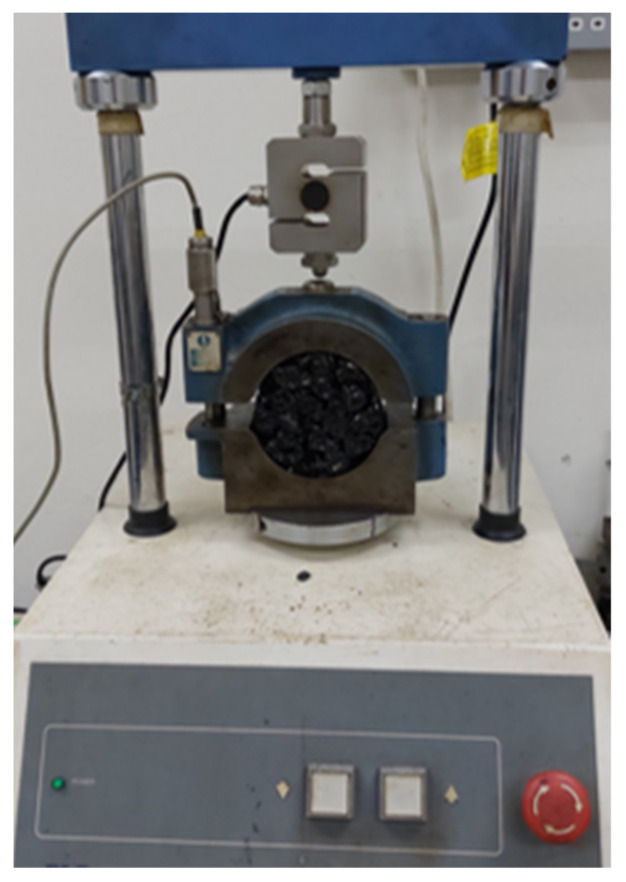
Marshall stability test set-up.

The Marshall stability test is defined as the maximum load that a Marshall compacted specimen can withstand before failure. The stability test was conducted in accordance with ASTM D6927 [57]. Prior to testing, the test specimens were conditioned in a water bath for 30 min at 60 °C of temperature. During the test, a load was applied at a constant rate of 50 mm/min until failure. The peak stability value (MS_dry_) was then recorded. The results of the three duplicate samples were averaged. The test set-up is shown in Figure 5.

#### 2.3.4. Compressive Strength Test

**Figure 6:**
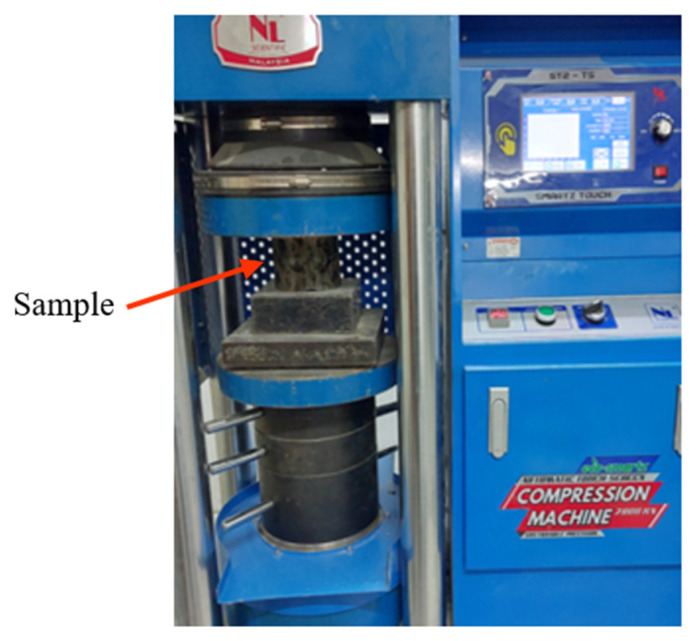
The test set-up of compressive strength.

The crack resistance performance of GlaSFlex composites can be effectively characterized by compressive strength as it is a good indicator of the overall quality of the composites under the loading conditions of heavy-duty trucks. The compressive strength test was carried out according to ASTM D1074 [58]. A load was applied at a rate of 0.50 kN/s using a 2000 kN compression machine until specimen failure, as shown in Figure 6. The compressive strength in dry conditions (CS_dry_) was calculated after testing.

#### 2.3.5. Dynamic Creep Test

**Figure 7:**
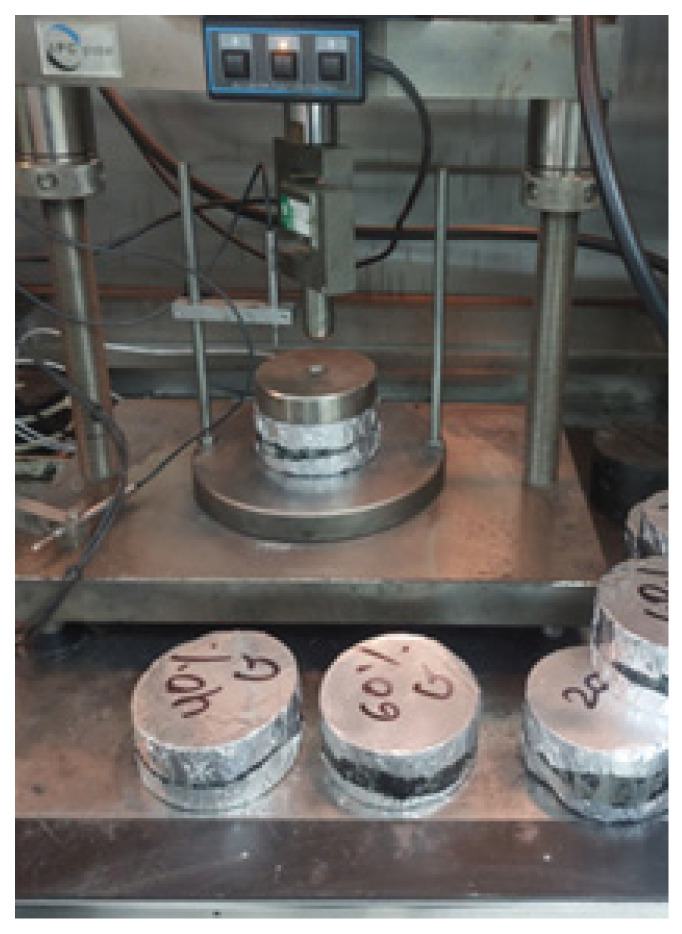
Samples in the dynamic creep testing chamber.

The dynamic creep test was conducted in accordance with AS 2891.12 [59] to determine the permanent deformation (rutting potential) of GlaSFlex pavement under repeated axial loading at a specified temperature. This was achieved by subjecting GlaSFlex specimens to thousands of loading cycles using a universal testing machine operating in stress-controlled mode to minimize the variance in sample cross-section. The Marshall test specimens were vertically cored using a diamond saw cutter to achieve a uniform thickness of 50 ± 1 mm. Each side of each specimen was coated with a thin layer of grease and graphite flakes to obtain a smooth surface, as illustrated in Figure 7. The test specimens were then conditioned in the machine chamber at 40 °C for 2 h before testing. As per AS 2891.12, each specimen was first subjected to a static preload of 20 kPa applied for one minute, and then followed by a dynamic stress of 200 kPa applied for 1 h. For each cycle, the load was applied for 0.5 s with a 1.5 s rest interval. The test was terminated upon reaching either 2800 cycles or 100,000 micro-strains, whichever occurred first.

#### 2.3.6. Indirect Tensile Stiffness Modulus Test

**Figure 8:**
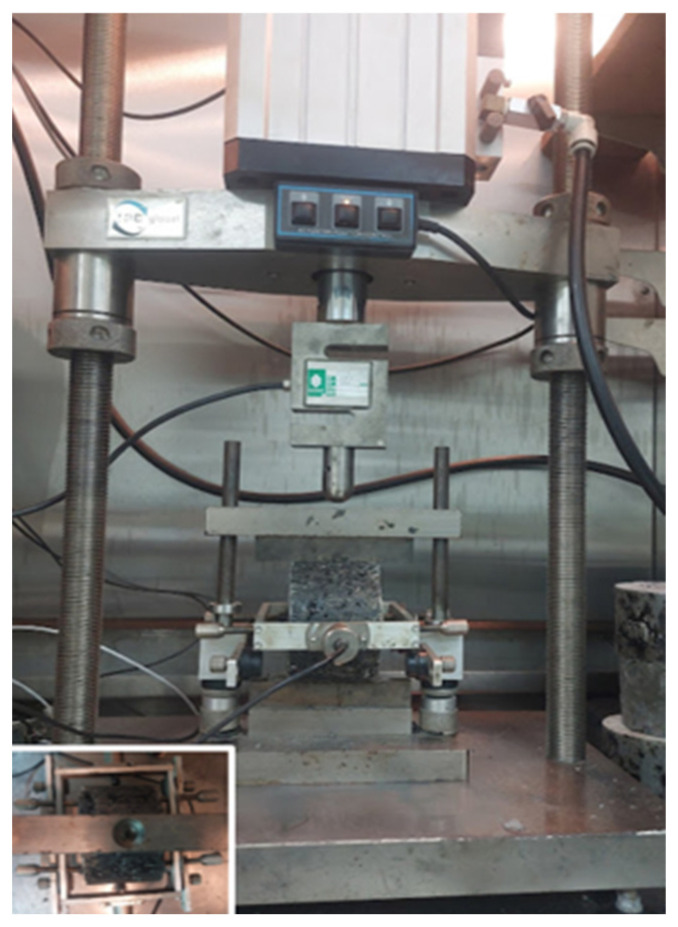
Sample set-up in the universal testing machine for ITSM measurement.

The ITSM test (BS 12697-26) [60] measures the distribution of traffic loads to the layers beneath the surface course, representing the long-term durability performance of the material. This test was conducted using a universal material testing machine. The Marshall test specimens were conditioned in the machine chamber at 25 °C for 2 h before the test is performed. Each specimen was tested at three various vertical loading points. During the test, the cylindrical specimen is positioned horizontally, as shown in Figure 8. The loading pressure head consists of two steel strips, approximately 12.7 mm wide, placed diametrically opposite on the specimen’s curved surface. These strips apply a vertical load, inducing indirect tensile stress perpendicular to the load direction. Deformation is measured using two horizontal displacement transducers placed at mid-height on opposite sides of the specimen.

### 2.4. Fuel Oil Corrosion Resistance Methods

**Figure 9:**
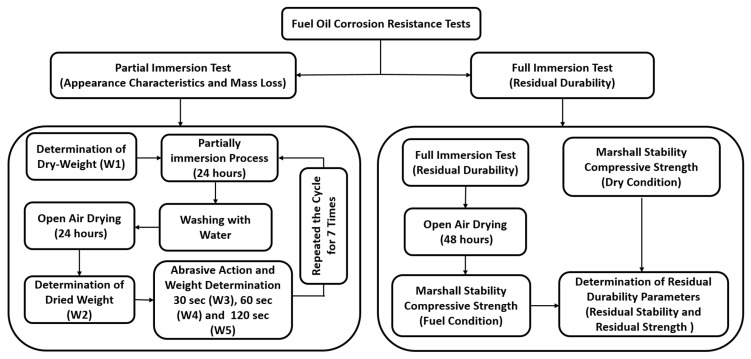
Diagram showing the procedure of oil corrosion resistance tests.

Pavement surfaces are frequently subjected to fuel oil spillage, particularly in industrial areas, gas stations, parking lots, and airports. This spillage often leads to various forms of pavement distress, such as raveling, stripping, and potholes. To investigate the effect of fuel oil exposure, two different investigation methods were adopted. For each method, the average value of three samples for each composite was calculated and recorded. Figure 9 demonstrates a schematic diagram detailing the fuel oil investigations.

#### 2.4.1. Partial Immersion (Appearance Characteristic and Mass Loss) Tests

The resistance to fuel oil corrosion after mass loss to partial immersion and abrasive actions was evaluated. As stipulated in BS-EN 12697-43 standard [61], there are two Parameters (A and B) that must be considered, as given in Equations (2) and (3), respectively. Parameter A signifies the mass loss due to immersion process in fuel oil, while Parameter B refers to the loss of mass due to abrasion actions. Additionally, a new Parameter (C) was adopted in a recent research study [62]. Parameter (C) represents the total mass loss due to the combined effect of abrasive action and immersion process and is calculated using Equation (4). Table 7 shows the fuel oil resistance of SFP composites as characterized by the BS 12697-43 standard and the Hofko criteria.

In this test, the dry weight of GlaSFlex composite specimens was recorded as W_1_. The specimens were then placed in a plastic container and partially immersed to a depth of 35 mm in RON95 for 24 h at room temperature. This method follows the procedure described in the BS-EN 12697-43 standard [61] and the method reported by Khan et al. [36]. After immersion, all specimens were taken out, washed with water, and left to dry in open air. After 24 h, the specimens were visually inspected, weighed, and recorded as W_2_. The specimens were then subjected to abrasive actions using a steel brush attached to a mechanical mixer. Finally, the weight of each specimen was recorded as W_3_, W_4_, and W_5_ after 30 s, 60 s, and 120 s of abrasive actions, respectively.

(2) $$A=\left[\frac{{W_{1}-W}_{2}}{W_{1}}\right]\times 100\%$$

(3) $$B=\left[\frac{{W_{2}-W}_{5}}{W_{2}}\right]\times 100\%$$

(4) $$C=\left[\frac{{W_{1}-W}_{5}}{W_{5}}\right]\times 100\%$$

#### 2.4.2. Full Immersion (Residual Durability) Tests

**Figure 10:**
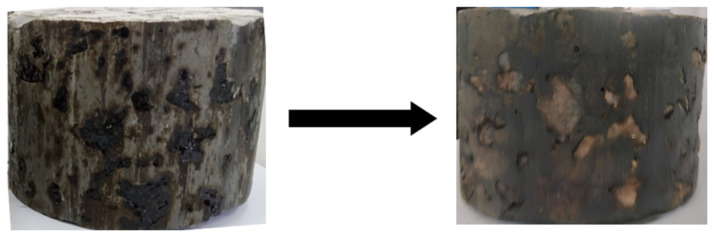
Appearance of GSP-00 composite specimen before and after fully immersion process in RON95 fuel oil.

In this investigation, each dry GlaSFlex specimen was first weighed and recorded as M1. The specimens were then placed in a cylinder-shaped metal bucket and fully immersed in an RON95 fuel bath for 24 h at room temperature. After immersion, the specimens were taken out, and RON95 was allowed to dry and evaporate for an additional 48 h. As presented in Figure 10, the specimens were applicable for further corrosion resistance investigations introduced later. This method was applied to all GlaSFlex composite test specimens. Finally, the corroded specimens were tested for Marshall stability and compressive strength.

The long-term fuel oil resistance and residual durability tests refer to the residual Marshall stability (RMS) test and residual compressive strength (RCS) test. The Marshall stability and compressive strength tests after full immersion in RON95 fuel oil were conducted, following the procedures described in Section 2.3.3 and Section 2.3.4, to determine the fuel-conditioned Marshall stability (MS_fuel_) and fuel-conditioned compressive strength (CS_fuel_). The RMS and RCS Parameters are defined by Equations (5) and (6), respectively.

(5) $$RMS=\left[\frac{{MS}_{fuel}}{{MS}_{dry}}\right]\times 100\%$$

(6) $$RCS=\left[\frac{{CS}_{fuel}}{{CS}_{dry}}\right]\times 100\%$$

## 3. Results and Discussion

Glasphalt mixtures were evaluated 3 days after preparation, while the GlaSFlex composites were tested after 28 days of impregnation. It is quite understandable that the SFP composites have higher rigidity and strength properties compared to the Glasphalt mixtures. Hence, the performance comparison was not made between Glasphalt mixtures before and after impregnation. Instead, evaluations focused on mixtures containing similar materials but varying FGA concentrations.

### 3.1. Air Voids

Table 8 demonstrates the air void results of the Glasphalt mixtures. The results showed that air content and porosity increased with the increasing FGA concentration. However, the air void values remain within the targeted limit (25–30%) for the design of the OGA layer used in an SFP mattress, as reported in the literature [63,64,65] and the REAM standard [40]. The enhancement in air voids is attributed to the fact that as the concentration of FGA increases, the draindown of the mastic material also increases. Compared to fine virgin aggregate, FGA has smoother surfaces and lower absorption capacity. These characteristics cause a higher proportion of the mastic material to drain out during production, which in turn increases the air content in the mixture. This phenomenon is well-documented, as the smoother surfaces reduce mechanical interlocking and absorption, allowing more mastic material to separate and drain.

Additionally, the hydrophobic nature of FGA significantly reduced the interfacial adhesion properties between the asphalt and FGA surface. The reduction in adhesion attributed to the poor wettability of FGA can be quantified by higher contact angle values, indicating weak surface compatibility between FGA and asphalt. This weak adhesion further contributes to reduced binder retention on the aggregate surface and promotes mastic drainage [12]. A recent research study has revealed that the contact angle of asphalt on FGA surfaces is significantly higher than on virgin aggregates [66], confirming the reduced affinity and its contribution to increased draindown and air voids. As such, the GOA-100 mixture had the highest porous percentage, with an increase of 6.5% compared to the GOA-00 mixture. A similar trend has been presented in other studies, which showed that adding FGA increases the air void rates of asphalt mixtures [67,68].

Table 8 also shows the air voids of the Glasphalt mixtures after impregnation. The grouting saturation results listed in Table 8 reveals that the GlaSFlex composites fulfilled the requirement (0.94–0.97) [65,66]. This trend attests to the interconnectivity of voids in the Glasphalt mixtures and the uniform distribution of the grout. Compared to the GSP-00 composite, the GSP-80 and GSP-100 composites had the highest saturation degrees because they initially had the highest voids before impregnation, which resulted in these voids being effectively filled by the glcement grout. A high residual void content (>6%) indicates poor impregnation that subsequently results in cracking of the following SFP mattress.

### 3.2. Marshall Stability

**Figure 11:**
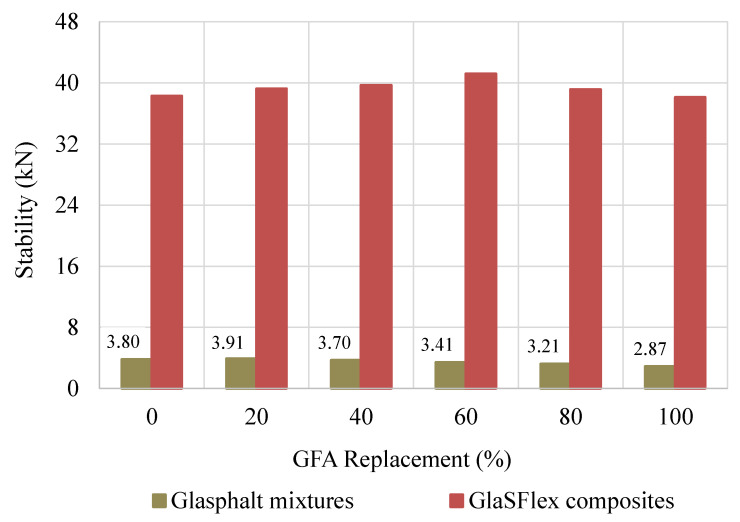
Effect of glass aggregate on stability before and after impregnation.

Marshall stability is a function of asphalt characteristics, air void rate, gradation, aggregate type and properties, and compaction efforts. The average stability values of both Glasphalt mixtures and GlaSFlex composites at different FGA concentrations are presented in Figure 11. With increasing FGA replacement, the stability of the Glasphalt mixtures decreased significantly, except the GOA-20, which has a slightly higher stability. The reduction in stability is mainly due to the high FGA concentration, as FGA tends to increase air void content and promote mastic draindown [3,12]. In addition, FGA has a cubical, flaky, elongated shape, and a smooth surface texture, resulting in inadequate interfacial adhesion properties, reduced interlocking, and lower internal friction compared to virgin aggregate [12,69,70].

This implies a reduction in the load-bearing capacity of the mixture. Previous studies have shown the adhesion property is mainly influenced by aggregate mineralogy, surface texture, and particle shape and size, as well as asphalt content [9,71]. As such, the lowest stability value was observed at 100% FGA replacement, representing a 24.4% decrease compared to the reference mixture

The Marshall stability of the GlaSFlex composites significantly improved after the voids were filled with grout, as portrayed in Figure 11. The results indicated that the stability values increased slightly with an FGA replacement up to 80%, and then a noticeable reduction in stability values was observed after that. As seen in Figure 11, the stability values of the SFP composites with FGA replacement were higher than that of the GSP-00 composite, except the GSP-100 composite. Compared to the GSP-00 composite, the GSP-60 composite achieved the highest value, with an increase of 7.6%. This is due to the enhanced impregnation resulting from the increased voids that were well filled with the grout. However, all GlaSFlex composites demonstrated high stability values, indicating strong resistance to cracking, surface deformation, and disintegration caused by heavy traffic loads and highly unfavorable environmental conditions. The increment in stability proposed a potential utilization of FGA as a fine aggregate in the OGA structural layer applied as an SFP mattress.

### 3.3. Compressive Strength

**Figure 12:**
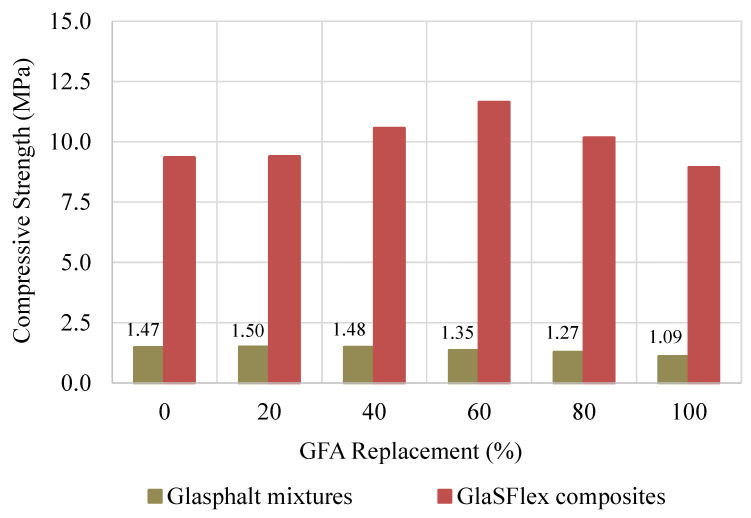
Effect of glass aggregate on compressive strength before and after impregnation.

It is important to ensure that Glasphalt mixtures have adequate strength before impregnation. The compressive strength results of Glasphalt mixtures and GlaSFlex composites are graphically plotted in Figure 12. With increasing FGA concentration, the strength of the Glasphalt mixtures decreased linearly. The compressive strength decreased with an increase in the FGA replacement until the lowest value was at 100% FGA, with a reduction of 25.7%. The trend can be attributed to excessive mastic material draindown, which results in increased air voids and the formation of a thin asphalt film around aggregates. This weakens the interfacial adhesion between the smooth surface of the FGA, the virgin aggregate, and the asphalt. In addition, FGA has lower hardness than fine virgin aggregate, thereby causing lower resistance to compressive forces. These combined physical characteristics contribute to the observed reduction in overall mixture strength.

The compressive strength of the GlaSFlex composites improved significantly due to the rigidity of the hardened glcement grout, as presented in Figure 12. There was a noticeable increment in compressive strength values with the FGA replacement up to 80%, followed by a slight reduction at 100% FGA. The improvement in compressive strength is attributed to the following: (i) the Glasphalt mixtures were better filled with the grout than the reference mixture because of higher air voids, and (ii) the presence of aluminum oxide and silicon dioxide in the RGA material, which enhanced the cohesion of the composites and improved resistance to compression [72,73]. However, all GlaSFlex composites exceeded the required minimum compressive strength (7 MPa). The highest value was observed at 60% FGA and increased by 24.7% when compared to the reference composite. The better the compressive strength, the longer the service life of the SFP mattress.

Based on the results of the Marshall stability and compressive strength tests, the Glasphalt mixtures have low bearing capacity and poor strength properties. These shortcomings and deficiencies can be attributed to several factors, including low asphalt content, high air voids, insufficient fine aggregate proportion, high draindown, and the inherent physical properties of FGA. These combined parameters contribute to premature pavement distresses such as cracking, potholing, aging, rutting, and raveling. Therefore, the results confirm that the OGA structural layer, regardless of FGA inclusion, is only suitable for use as an SFP mattress. It is not recommended for use as a porous asphalt-wearing course, especially under wet conditions. Consequently, the fuel oil corrosion resistance of Glasphalt mixtures was not investigated in this study.

### 3.4. Appearance Characteristics

**Figure 13:**
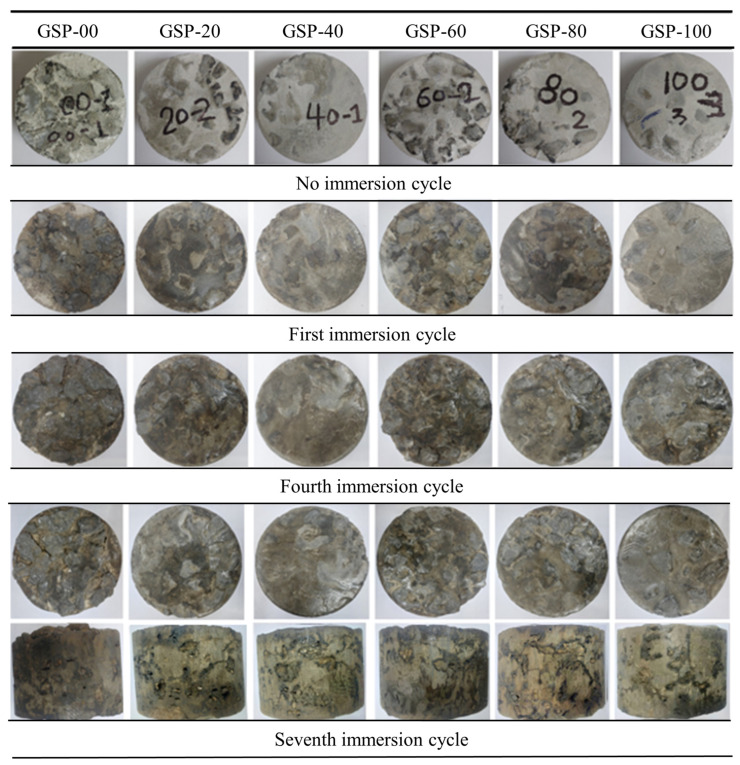
Appearance changes in GlaSFlex composites after exposure to fuel oil, showing visible damage characterized by small holes with diameters less than 2 mm.

All GlaSFlex test samples were visually inspected, and the change in the appearance of the composite samples after the first, fourth, and seventh immersion cycles are presented in Figure 13. A very small amount of mastic material, mainly consisting of filler and asphalt, began to strip from the surfaces, sides, and edges of the composites after the first immersion cycle. GlaSFlex composite samples showed notable differences in appearance at this cycle phase. However, the structural integrity of the samples remained largely intact. As the cycles progressed, further changes were evident; however, these were less pronounced after the fourth cycle, as most of the mastic had already been stripped from the surfaces, sides, and edges. By the seventh immersion cycle, all composite samples displayed varying degrees of surface damage due to prolonged fuel oil exposure and abrasive actions. Small holes and gaps were particularly noticeable along the sides that were not coated with glcement grout, as illustrated in Figure 13. A similar trend was reported by Khan et al. [36], which showed that incorporating waste PET as an additive significantly reduced the detrimental effects of fuel oil on the surface appearance of SFP.

### 3.5. Mass Loss

**Figure 14:**
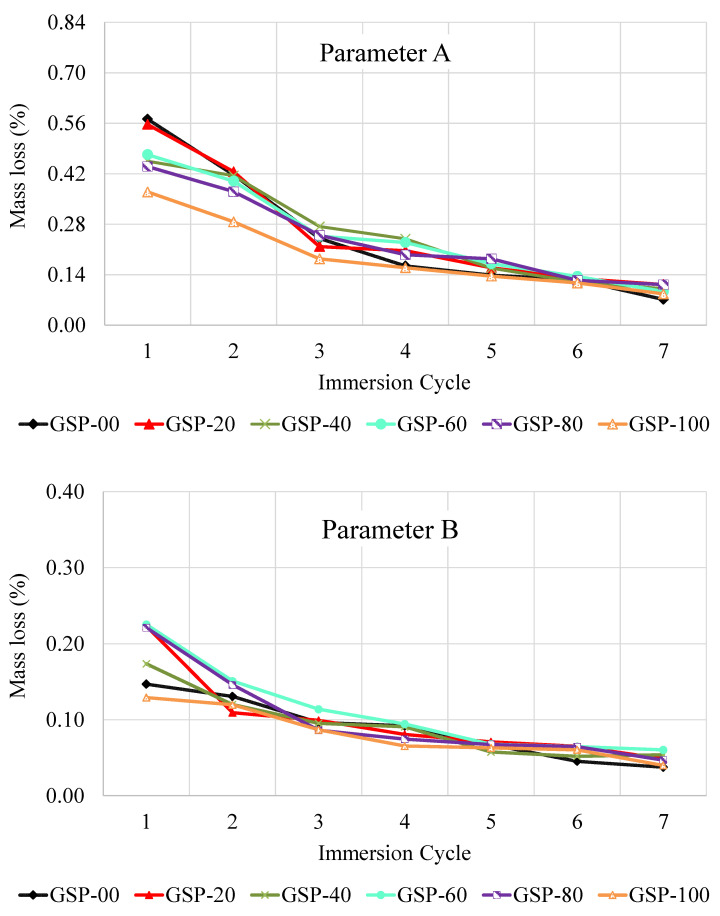
Parameter A and Parameter B representing the fuel oil corrosion resistance of GlaSFlex composites.

**Figure 15:**
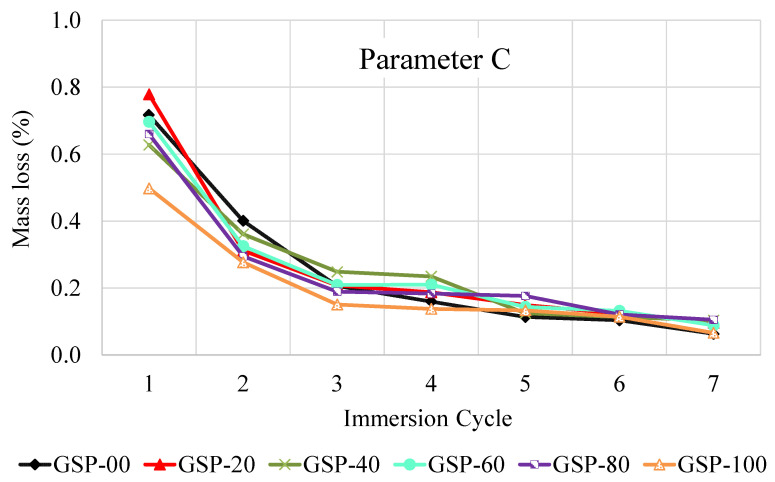
Parameter C representing fuel oil corrosion resistance of GlaSFlex composites.

The mass loss of GlaSFlex composites due to fuel oil corrosion and abrasive actions is presented in Figure 14 and Figure 15. Parameter A shown in Figure 14 denotes the effect of oil corrosion and washing, while Parameter B refers to the effect of abrasive actions on the GlaSFlex samples. The results revealed significant resistance to fuel oil corrosion “RON95 gasoline”, and there was negligible mass loss following the immersion period, as presented in Figure 14 (Parameter A). The mass loss of all GlaSFlex composites decreased gradually with the oil immersion period. A noticeable reduction in mass loss values occurred after the third immersion cycle. In addition, all GlaSFlex composites showed a negligible increase in loss of mass with an increasing number of immersion cycles. However, the GSP-20 composite exhibited a slightly higher mass loss than the others after the last cycle.

Similarly, the mass loss following abrasive actions was negligible with increasing immersion cycles. As can be seen in Figure 14 (Parameter B), all GlaSFlex composites exhibited a mass loss rate of less than 1%, indicating excellent abrasion resistance. Beyond the fifth cycle, the mass loss values of the composites did not accelerate significantly with additional exposure cycles, remaining relatively similar. This is because of the smoothing of the sample surfaces due to abrasion, as displayed in Figure 13. The mass loss of the different composites was found to be close to each other between the fifth cycle and the seventh cycle. According to Parameter B, the composites have a high resistance to mass loss due to abrasive actions, indicating that impregnation with the glcement grout was appropriate and feasible. By considering Parameters A and B in line with BS requirements [48], it is evident that the GlaSFlex composites have excellent resistance to fuel oil spillage. Notably, the highest recorded mass loss did not exceed 0.60% for Parameter A and 0.25% for Parameter B.

The fuel oil resistance results of GlaSFlex composites to the combined effect of abrasive action and immersion (Parameter C) are presented in Figure 14. The results showed that different GlaSFlex composites displayed similar behavior and comparable values. After the seventh immersion cycle, the mass loss for all composites was negligible (about 0.66%). By examining Parameter C, the composites clearly showed high fuel oil resistance, as the results of Parameter C did not exceed 0.8%. The lowest total mass loss after the seventh cycle was recorded at 1.4% for the mixture containing 100% FGA, compared to 1.7% for the reference mixture. These results confirm that asphalt mixtures incorporating various FGA replacement levels exhibited high and interconnected voids that were fully filled with the glcement grout, as observed at the interfaces. This structural feature effectively reduced the effect of fuel oil corrosion. Therefore, FGA can be considered a viable full replacement to fine aggregate in the production of the OGA structural layer used in an SFP mattress.

Referring to the mass loss criteria [59,60] presented in Table 7, the low mass loss value indicates the SFP composite has high resistance to fuel oil corrosion. Based on the results shown in Figure 13 and Figure 14, GlaSFlex composites showed superior resistance to oil-induced corrosion. This trend means that GlaSFlex composites can be successfully used in areas prone to fuel oil spillage. Similar results of mass loss for SFP composites utilizing irradiated waste PET-based grouts were reported by Khan et al. [36].

### 3.6. Dynamic Creep

**Figure 16:**
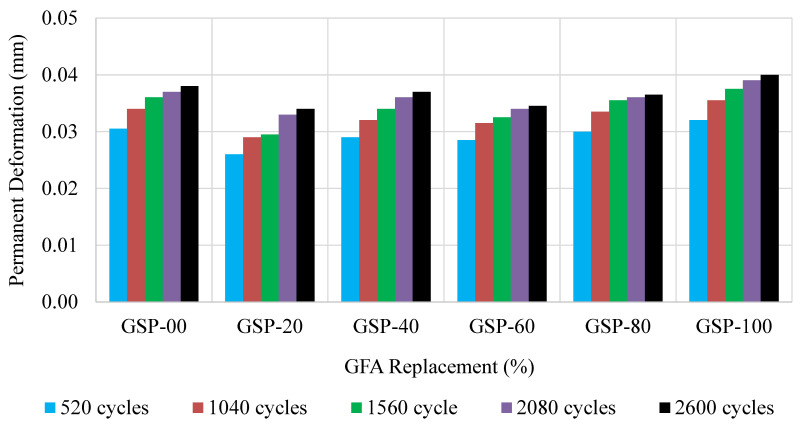
Permanent deformation of SFP composites at different glass concentrations.

Dynamic creep simulates the longitudinal depressions in wheel paths, commonly known as permanent deformation. In this study, the performance of GlaSFlex composites was evaluated at five cycle intervals to evaluate their resistance to permanent deformation at different life periods. The results for each interval are presented in Figure 16.

Figure 16 demonstrates a consistent trend of increasing permanent deformation, as the number of load cycles increased. The results showed that the most significant deformation occurred during the initial loading cycles, and then, a gradual increase in deformation. This trend indicates that the GlaSFlex composites closely resemble flexible pavement materials [11,74]. However, GlaSFlex composites exhibited high rutting resistance, with the maximum deformation not exceeding 0.045 mm. The SFP composites with FGA replacement had lower rut depths than the reference composite, except the GSP-100 that displayed higher deformation at 0.04 mm compared to the deformation of GFP-00 (0.038 mm). The improved rutting resistance is attributed to the use of FGA as a fine aggregate replacement. Its pozzolanic material, which contains reactive components such as aluminum oxide and silicon dioxide [64], increased the bonding between the composite compositions, thereby improving its durability [61,62]. Additionally, the increased grout content in the GlaSFlex composites promoted the strength and deformation resistance [11]. The results are agreement with the results reported by Afonso et al. [75] and Huynh [76], further supporting the efficacy of GlaSFlex composites in mitigating permanent deformation.

### 3.7. Indirect Tensile Stiffness Modulus

**Figure 17:**
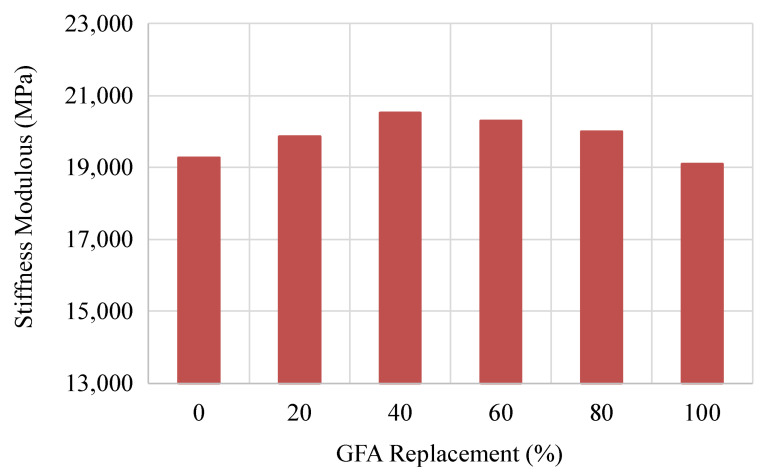
Stiffness modulus of SFP composites at different glass concentrations.

The ITSM was determined for GlaSFlex composites to assess their long-term durability performance. Figure 17 shows the ITSM of the composites. The stiffness modulus values of GlaSFlex composites were relatively similar as the level of FGA replacement increased. Compared to the reference composite, the highest stiffness modulus value was achieved at 40% FGA, which had a slightly higher increase (6.6%). The enhancement in the stiffness is due to better grout penetration and interlocking in the GOA-40 mixture. The higher FGA concentration led to increased air voids in the Glasphalt mixture, which allowed the grout to fill the voids more effectively, resulting in a denser and stiffer composite. This, in turn, confirms that the glcement grout was well distributed in the composites. Inclusion of more FGA decreased the convenience adhesion properties, deteriorating the stiffness. The ITSM values are in agreement with the Marshall stability and compressive strength values of the GlaSFlex composites.

The stiffness of the GlaSFlex composites also shows that their structural behavior more closely resembles that of asphalt pavement materials rather than concrete pavement materials, indicating that the composites exhibit viscoelastic characteristics. Oliveira [74] revealed that the stiffness of SFP composites decreases with increasing the temperature, further confirming the behavior of SFP composites as viscoelastic materials. However, GlaSFlex composites had a high stiffness modulus. This means the GlaSFlex composites have superior structural behavior, confirming the open-graded glass asphalt mixture can be used as an SFP mattress in high-load applications, such as airport runways and bus station platforms. Similar behaviors have been observed in previous studies, which also showed that SFP composites have high strength and stiffness modulus [22,77].

### 3.8. Residual Marshall Stability

**Figure 18:**
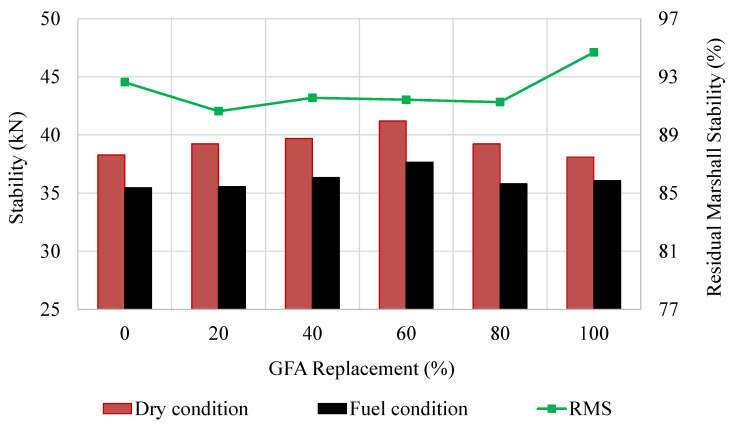
Residual Marshall stability of SFP composites at different glass concentrations.

The RMS after exposure to fuel oil corrosion is another method of investigating the resistance of GlaSFlex composites against fuel oil spillage. There is no specific standard to determine the materials’ resistance to fuel oil corrosion by this method. For each condition, the Marshall stability analysis results are portrayed in Figure 18. The effect of RON95 gasoline on the stability of GlaSFlex composites was negligible. After 24 h of immersion in an RON95 bath, the composites retained high Marshall stability values. The result indicates that the interconnected voids in the Glasphalt mixtures were effectively saturated with the glcement grout, and therefore the RON95 was unable to penetrate the exterior structure of the composites. 

As depicted in Figure 18, the RMS values of the GlaSFlex composites were close to 91%, except the GSP-100 composite had a slightly higher value (2.2%). This can be attributed to the fact that the fuel-conditioned Marshall stability of composites with different FGA concentrations were higher compared to the GSP-00 composite. However, no considerable differences were observed in the RMS values of the various GlaSFlex composites. This, in turn, shows the GlaSFlex composites exhibit high durability characteristics and crack resistance, even under harsh environmental conditions such as exposure to fuel oil, deicing salting, alkaline rain, and acid rain that are the most obvious enemies of asphalt.

Although the Marshall stability of the reference SFP composite reduced slightly from 38.27 kN under dry conditions to 35.45 kN after exposure to fuel, it still retained a high RMS of 92.62%, indicating strong resistance to fuel oil corrosion. In contrast, the reference asphalt mixture experienced a significant reduction in stability after 24 h of immersion in an engine oil bath, dropping from 3.80 kN to 2.59 kN, retaining only 68.11% of its original stability. In short, the GlaSFlex composites demonstrated superior performance against fuel oil spillage. Similar trends in RMS were observed in previous studies, with Li et al. [24] reporting an RMS of 72.6% for asphalt mixture, and Hao et al. [38] documenting a higher RMS of 89% for SFP composite.

### 3.9. Residual Compressive Strength

**Figure 19:**
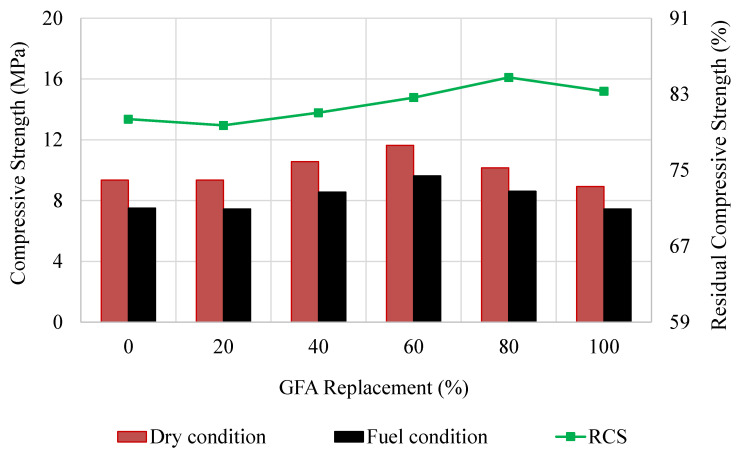
Residual compressive strength of SFP composites at different glass concentrations.

The RCS parameter is a reliable indicator of the durability and resistance of SFP composites to deterioration and abrasive action under harsh conditions. The compressive strength results of GlaSFlex composites for different conditions are shown in Figure 19. The results after full immersion in RON95 gasoline showed that the composites exhibited behavior similar to that observed before immersion; additionally, all values were above the targeted value (7 MPa) prescribed by REAM [40]. This is a good indication for the application of GlaSFlex composites.

The RCS values increased with the increment in the FGA replacement. The GSP-80 and GSP-100 composites had the highest RCS values by an increase of 5.6% and 4.4%, respectively, in comparison to the GSP-00 composite. This improvement is attributed to higher grout saturation levels, as listed in Table 8, which enhanced resistance to fuel oil corrosion and reduced mastic stripping caused by RON95, thereby the interfacial adhesion properties were not affected. Hence, it can be concluded that the SFP composite at different FGA replacement levels demonstrates superior durability performance and high crack resistance under heavy traffic loads, even in the presence of petroleum product spillage, due to its components and high density.

### 3.10. Microstructure

Microstructural characterization is a critical analytical approach in experimental research for elucidating the micromorphology and interfacial bonding behavior of constituent materials, which directly governs the mechanical and durability performance of GlaSFlex composites. FESEM offers superior spatial resolution and depth of field, enabling the high-fidelity imaging of surface and subsurface features. In this study, FESEM was employed to investigate the interfacial morphology and elemental distribution within the GlaSFlex composite, providing comprehensive insights into the microstructural integration between the glcement grout and Glasphalt mixture.

**Figure 20:**
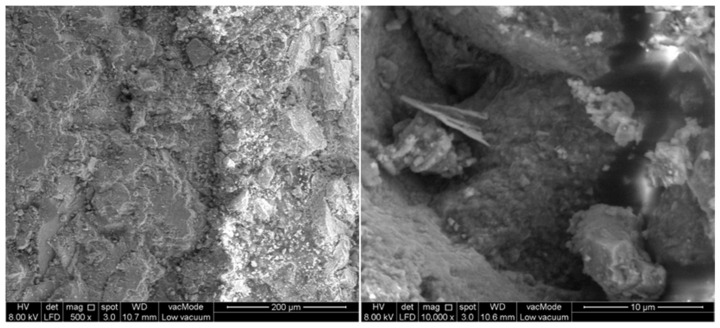
Microstructure image of SFP composite containing glass.

Figure 20 presents the FESEM micrographs of the GlaSFlex composite, revealing the internal morphology and micromechanical characteristics. The images clearly show the propagation and distribution of the glcement grout within the GlaSFlex structure. The grout visibly emerges and extends along the interface with the Glasphalt mixture, while the FGA is not, indicating a strong interfacial bond throughout the full depth of the composite structure. This confirms the effective integration between the glcement grout and the Glasphalt mixture.

The microstructural evaluation also highlights several favorable macro-properties of the GlaSFlex composite, including increased surface roughness, high density, enhanced interfacial adhesion, and significantly reduced porosity. These characteristics contribute to superior resistance to cracking and rutting, improved compressive strength, and reduced water absorption that are key indicators of its suitability for SFP applications. Furthermore, replacing fine virgin aggregate with FGA in cement grout contributes to the matrix densification through the formation of amorphous and semi-crystalline calcium silicate hydrate [78], resulting in a more robust composite structure. From a microscopic perspective, the GlaSFlex composite exhibits a durable and well-optimized formulation, which enhances its macro-scale mechanical performance and contributes to superior structural integrity.

## 4. Conclusions

In this research, SFP composites prepared at six different FGA concentration levels were evaluated in terms of mechanical performance properties, appearance characteristics, mass loss, and fuel oil resistance. The following conclusions can be drawn:

1. The Glasphalt mixtures had air contents within the targeted limit. Similarly, the degree of grout saturation ranged from 94% to 97%, indicating well-interconnected air voids.
2. The Marshall stability of the Glasphalt mixtures decreased, as the FGA replacement level increased. In contrast, the GlaSFlex composites exhibited higher Marshall stability compared to the reference composite. A similar improvement was observed in the ITSM results, further confirming the enhanced mechanical performance of GlaSFlex composites. Additionally, these composites displayed lower permanent deformation than the reference composite, indicating enhanced resistance to rutting.
3. The reference mixture had a higher compressive strength than the Glasphalt mixtures. On the other hand, the compressive strength of the GlaSFlex composites were higher when compared to the reference composite. All compressive strength values were above the minimum allowable limit (7 MPa).
4. One of the most significant features of the GlaSFlex composites is their strong resistance to fuel oil corrosion. The composites demonstrated high durability, low mass loss rate (<1%), and minimal degradation upon exposure to gasoline. At 100% FGA concentration, the composite achieved a compressive strength of 8.93 MPa, a Marshall stability over 38 kN, a stiffness modulus of 19,091 MPa, a residual stability of 94.7%, and a residual compressive strength of 83.3%. These results show the suitability of GlaSFlex composites for use in heavy duty, fuel-exposed areas such as bus terminals, airports, gas stations, and industrial floors.
5. The use of FGA in SFP construction projects offers a sustainable solution to the environmental challenges associated with GW disposal. GlaSFlex composite presents an economical and practical alternative, as it not only extends the service life of landfills but also reduces the demand for virgin aggregate materials. Additionally, the use of FGA in SFP construction can contribute to longer-lasting pavements with lower maintenance needs, which reduces life-cycle costs.

## Figures and Tables

**Table 1:** Physical properties of FGA and granite aggregates.

Property	Soundness (%)	Water Absorption (%)	Specific Gravity	Abrasion Mass (%)	Crushing Value (%)
Reference	ASTM C88 [41]	BS 812 Part 107 [42]	BS 812 Part 107 [42]	ASTM C131 [43]	BS 812 Part 110 [44]
FGA	6.22	0.59	2.47	-	-
Coarse granite	4.27	1.08	2.55	19.48	21.70
Fine granite	3.26	0.92	2.58	-	-

**Table 2:** Aggregate gradation of open-graded glass asphalt mixtures.

Sieve Size (mm)	Limits of Passing (%)			Retained Weight (%)
	Lower	Upper	Mix	
25	100	100	100	0
20	95	100	95	5
14	4	18	17	78
8	4	14	13	4
4.75	4	12	10	3
2	4	10	6	4
0.075	3	5	4	2

**Table 3:** Basic physical properties of asphalt binder.

Property	Softening Point	Penetration at 25 °C	Ductility at 25 °C	Viscosity at 135 °C
Reference	ASTM D36 [45]	ASTM D5 [46]	ASTM D113 [47]	ASTM D4402 [48]
Value	51.76 °C	64.90 mm	107.40 cm	493.68 mPa.s

**Table 4:** Basic physical properties of cement.

Property	Specific Gravity	Setting Time (minute)		Compressive Strength (N/mm^2^) at 28 Days
		Initial	Final	
Reference	ASTM C188 [49]	ASTM C191 [50]		ASTM C109 [51]
Value	3.11	117	306	42.09

**Table 5:** Glass and granite concentrations in open-graded asphalt mixtures.

Mix Design	Aggregate (%)			Glasphalt	GlaSFlex
	Fine Granite	Coarse Granite	FGA		
Mix with 0% FGA	100	100	00	GOA-00	GSP-00
Mix with 20% FGA	80	100	20	GOA-20	GSP-20
Mix with 40% FGA	80	100	40	GOA-40	GSP-40
Mix with 60% FGA	40	100	60	GOA-60	GSP-60
Mix with 80% FGA	20	100	80	GOA-80	GSP-80
Mix with 100% FGA	00	100	100	GOA-100	GSP-100

**Table 6:** Contents of glcement grout and properties.

Parameter	Value	Property	Value
Maximum grain size (mm)	0.30	Flow out	11.8 s
Water/cement ratio	0.26	Density	2.15 g/cm^3^
Sand/cement ratio	0.30	Flexural strength at 28-day	13.9 N/mm^2^
Superplasticizer/cement ratio	0.025	Compressive strength at 28-day	121.8 N/mm^2^

**Table 7:** Mass loss criteria.

Resistance	Good	Moderate	Poor
BS 12697-43 criteria	A ≤ 5% and B < 1%	A ≤ 5% and 1% ≤ B ≤ 5%	A > 5% and B > 5%
Hofko criteria	C < 6%	6% ≤ C ≤ 10%	C > 10%

**Table 8:** Effect of glass aggregate on air voids in asphalt mixture before and after impregnation.

Mix	Air Void (%)	Degree of Saturation (%)
Mix with 0% FGA	26.1	0.96
Mix with 20% FGA	26.0	0.96
Mix with 40% FGA	26.0	0.96
Mix with 60% FGA	26.7	0.96
Mix with 80% FGA	27.6	0.97
Mix with 100% FGA	27.8	0.97

## Data Availability

The raw data supporting the conclusions of this article will be made available by the authors on request.

## Abbreviations

FESEM	field emission scanning electron microscopy
FGA	fine glass aggregate
GlaSFlex	glass-semi-flexible pavement
Glasphalt	open-graded glass asphalt
Glcement	glass cement
GW	glass waste
ITSM	indirect tensile stiffness modulus
OGA	open-graded asphalt
OPC	ordinary Portland cement
RCS	residual compressive strength
REAM	Road Engineering Association of Malaysia
RMS	residual Marshall stability
SFP	semi-flexible pavement
